# High Flux Nanofiltration Membranes with Double-Walled Carbon Nanotube (DWCNT) as the Interlayer

**DOI:** 10.3390/membranes12101011

**Published:** 2022-10-19

**Authors:** Zhen Wang, Xiaojuan Wang, Tao Zheng, Bing Mo, Huacheng Xu, Yijun Huang, Jian Wang, Congjie Gao, Xueli Gao

**Affiliations:** 1 Frontiers Science Center for Deep Ocean Multispheres and Earth System, Key Laboratory of Marine Chemistry Theory and Technology, Ministry of Education, College of Chemistry and Chemical Engineering, Ocean University of China, Qingdao 266100, China; 2 SEPCOIII Electric Power Construction Co., Ltd., Qingdao 266100, China; 3 Quanzhou Lanshen Environmental Protection Research Institute Co., Ltd., Quanzhou 362000, China; 4 The Institute of Seawater Desalination and Multipurpose Utilization, SOA, Tianjin 300192, China

**Keywords:** interfacial polymerization, nanofiltration membranes, double-walled carbon nanotube, interlayer

## Abstract

Nanofiltration (NF) membranes with a high permeability and rejection are of great interest in desalination, separation and purification. However, how to improve the permeation and separation performance still poses a great challenge in the preparation of NF membranes. Herein, the novel composite NF membrane was prepared through the interfacial polymerization of M-phenylenediamine (MPD) and trimesoyl chloride (TMC) on a double-walled carbon nanotube (DWCNT) interlayer supported by PES substrate. The DWCNT interlayer had a great impact on the polyamide layer formation. With the increase of the DWCNT dosage, the XPS results revealed an increase in the number of carboxyl groups, which decreased the crosslinking degree of the polyamide layer. Additionally, the AFM results showed that the surface roughness and specific surface area increased gradually. The water flux of the prepared membrane increased from 25.4 L/(m^2^·h) and 26.6 L/(m^2^·h) to 109 L/(m^2^·h) and 104.3 L/(m^2^·h) with 2000 ppm Na_2_SO_4_ and NaCl solution, respectively, under 0.5 MPa. Meanwhile, the rejection of Na_2_SO_4_ and NaCl decreased from 99.88% and 99.38% to 96.48% and 60.47%. The proposed method provides a novel insight into the rational design of the multifunctional interlayer, which shows great potential in the preparation of high-performance membranes.

## 1. Introduction

With population growth and economic development, fresh water scarcity has become extremely serious [1,2,3,4,5], and 25% of the population currently do not have enough fresh water. Due to low energy requirements, good stability and environment compatibility, membrane separation technology has been widely applied to alleviate the shortage of water resources. As a kind of pressure-driven membrane separation technology [6], nanofiltration (NF) with high permeability and selectivity for specific components has developed rapidly in recent years. Until now, NF has been widely applied in water treatment [7,8,9,10,11,12], biomedicine [9,13], food [14,15,16,17] and environmental protection [18,19,20,21]. The preparation methods of NF membranes mainly include phase-transformation [22], surface coating [23], layer-by-layer self-assembly [24], surface grafting [25], co-extrusion [26] and interfacial polymerization [27]. Among the above methods, interfacial polymerization has the advantages of a simple operation and low cost; therefore, it is the most commonly used method in the preparation of commercial NF membranes.

Currently, there are many studies on the preparation of NF membranes by the interfacial polymerization method, most of which focus on the preparation of substrate and the selection of reaction monomers and additives. The studies related to substrate indicate that the pore structure and properties (pore diameter, porosity, hydrophilicity, etc.) of substrate can directly affect the diffusion rate of monomer in the interfacial polymerization reaction and then change the morphology and properties of polyamide layers [28,29,30,31]. Misdan et al. [29] used PS, PES and PEI membrane as the substrate and indicated that the enhanced hydrophilicity of substrates could reduce the crosslinking degree of polyamide layers and then improve the permeability of NF membranes. Other related literatures have studied interfacial polymerization monomers. Hydrophilic monomers can improve the surface hydrophilicity of NF membranes and thus increase the water flux [32,33,34,35]. For example, Ma et al. [34] synthesized zwitterionic monomers using PEI and SBMA, and then prepared NF membranes through the interfacial polymerization method. The results showed that the water flux of NF membranes was over two times that of the membranes without zwitterion. In addition to hydrophilic monomers, monomers with special functional groups can also improve the performance of NF membranes [36,37,38].

With the development of nanomaterials, researchers have turned their attention to the modification of NF membranes using nanostructured materials. Some studies reported that the addition of nanomaterials (carbon nanotubes, graphene oxide, metal organic frameworks, covalent organic frameworks, etc.) into aqueous solution or oil phase solution could change the structures and properties of polyamide layers [39,40,41,42,43,44,45,46,47]. Zhao et al. [40] utilized a hydrophilic carbon nanotube (CNT) to increase the water flux of NF membranes without much decrease in salt rejection, and thus broke through the “trade-off” effect. Similarly, Zhu et al. [44] added a metal organic framework (MOF) into the polyamide layer to improve the surface roughness of NF membranes. The results showed that the permeability of NF membranes was greatly enhanced under the premise of maintaining salt rejection. Furthermore, some studies used nanomaterials as an interlayer to improve the properties of membranes [48,49,50,51,52,53,54]. Wang et al. [50] coated cellulose nanocrystals on the substrate surface as an interlayer, which decreased the cross-linking degree of the polyamide layer by regulating the interfacial polymerization process, and thus improved the water permeation. Wu et al. [53] modified NF membranes with PDA-COFs and reduced the thickness of the polyamide layer from 79 nm to 11 nm. The study indicated that the NF membranes simultaneously had a high water flux and salt rejection.

The double-walled carbon nanotube (DWCNT) possesses a good hydrophilicity and interconnected structure, which has the potential to endow NF membranes with a perfect performance. In this study, DWCNT was modified with dopamine hydrochloride and then used as an interlayer to improve the performance of NF membranes. The results showed that the prepared membrane had a high water flux and rejection of Na_2_SO_4_. It is expected that the study will be able to facilitate the preparation of NF membranes with an enhanced permeability, and thus it shows great potential in the industrial preparation.

## 2. Materials and Methods

### 2.1. Materials

Commercial PES membranes (Haining Zhengda Filtration Equipment Co., Ltd., Haining, China) with a pore size of 100 nm were used as substrate for the preparation of NF membranes. The COOH-functionalized DWCNT (60%, Nanjing XFNANO Materials Tech Co., Ltd., Nanjing, China) with an inner diameter of 1–3 nm, outer diameter of 2–4 nm and length of 0.5–2 μm, dopamine hydrochloride (98%, Aladdin, Vienna, VA, USA), sodium dodecyl benzenesulfonate (SDBS, 98%, Aladdin) and Tris-HCl (1 M, Ph = 7.5, Aladdin) were used to modify the substrate. M-phenylenediamine (MPD, 99.5%, Aladdin), trimesoyl chloride (TMC, 99.5%, Aladdin), sodium dodecyl sulfate (SDS, 99%, Aladdin), camphor sulfonic acid (CSA, 99%, Aladdin), triethylamine (TEA, 99.5%, Aladdin), N-hexane (99%, Aladdin) and deionized water (18 MΩ) were used to fabricate NF membranes by the interfacial polymerization method. NaCl (99.5%, Tianjin Fengchuan Chemical Reagent Co., Ltd., Tianjin, China) and Na_2_SO_4_ (99.5%, Tianjin Fengchuan Chemical Reagent Co., Ltd., Tianjin, China) were used to test the performance of the NF membrane.

### 2.2. Modification of the Substrate with DWCNT

Dopamine hydrochloride was used to modify DWCNT, in order to make DWCNT stably adhere to the substrate surface [49]. A total of 10 mg DWCNT and 100 mg SDBS were dispersed in 100 mL deionized water, and the mixed solution was subjected to sonication for 10 h. Then, the obtained dispersion was centrifuged at 10,000 rpm for 1 h to remove the undispersed DWCNT. The concentration of DWCNT in dispersion was about 0.06 mg/mL. After that, we added 10 mg dopamine hydrochloride into 100 mL DWCNT dispersion and stirred for 1 h at 40 °C. Then, we added 10 mL Tris-HCl into the mixture and reacted it for at least 36 h. Finally, the solution was centrifuged at 8000 rpm for 30 min to obtain the polydopamine/DWCNT dispersion. In this study, different dosages of polydopamine/DWCNT dispersion (0 mL, 1 mL, 3 mL, 5 mL, 7 mL and 9 mL) were diluted to 100 ml and then adhered to the PES membrane after suction filtration, which were named UF-0, UF-1, UF-3, UF-5, UF-7 and UF-9, respectively. The modified PES membranes were dried for 5 min at 80 °C and then stored in deionized water for later use.

### 2.3. Preparation of NF Membranes

**Figure 1:**
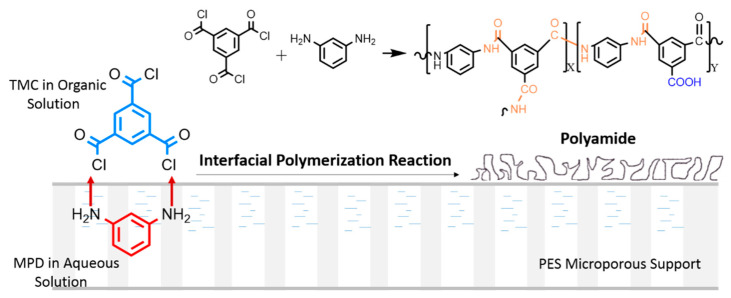
Schematic representation of the interfacial polymerization reaction between MPD and TMC at the surface of PES substrate.

The interfacial polymerization process is depicted in Figure 1. First of all, the PES membranes were fixed on a clean glass plate with the dense layer facing up, then 2.0 wt% MPD aqueous solution containing 2.6 wt% CSA, 1.1 wt% TEA and 0.1 wt% SDS was poured onto the top surface of PES membranes for 1 min, followed by removing excess MPD solution by a rubber roller. After that, 0.15 wt% TMC/n-hexane solution was poured onto the MPD-impregnated side of the PES substrate for 40 s, and the polyamide formed through interfacial polymerization reaction. Then, the membranes were rinsed thoroughly with pure n-hexane to remove unreacted TMC. Finally, the membranes were put into the oven (80 °C) for 5 min and soaked in deionized water for later use. The NF membranes were named NF-0, NF-1, NF-3, NF-5, NF-7 and NF-9 according to the substrate membranes, respectively.

### 2.4. Membranes Characterization

Attenuated total reflection Fourier transform infrared spectroscopy (ATR-FTIR, Nicolet IS50, Thermo Scientific, Waltham, MA, USA) can analyze the information of functional groups according to characteristic peaks of the infrared spectrum, and thus confirm the formation of the polyamide layer [55]. X-Ray photoelectron spectroscopy (XPS, K-Alpha, Thermofisher Scientific, Waltham, MA, USA) was used to characterize the chemical components and functional groups of the membranes surface. The surface morphology and properties (including roughness, surface area and projected area) of membranes were characterized by an atomic force microscope (AFM, AIST, Tokyo, Japan). Additionally, the specific surface area could be calculated by the Equation (1). The contact angle was measured by the static contact angle measuring instrument (DSA100, Berlin, Germany), which indicated the membranes’ surface hydrophilicity. The zeta potential instrument (SurPASS 3, Anton Paar, Berlin, Germany) was used to analyze the electric charge of membranes [50].

(1) $$\mathrm{Specific}\;\mathrm{surface}\;\mathrm{area}=\frac{\mathrm{Surface}\;\mathrm{Area}}{\mathrm{Projected}\;\mathrm{Area}}$$

### 2.5. Membrane Filtration Performance

The filtration performance of NF membranes was tested in a lab-scale cross-flow filtration system with an effective area of 28.26 cm^2^ at 25 ± 0.5 °C and 0.5 MPa. In this study, the membranes were pre-compacted for 1 h under 0.5 MPa with deionized water, and then the performance of membranes was tested by replacing deionized water with 2000 ppm NaCl solution and 2000 ppm Na_2_SO_4_ solution, respectively. The water flux and salt rejection were calculated by the following equation [4,56]:

(2) $$F=\frac{V}{A\times \Delta t}$$

where *F* is the water flux of membranes, L/(m^2^·h); *V* is the volume of permeation during the experiment, L; *A* is the effective area of the membrane, m^2^; and Δt is the operation time, h.

(3) $$R=\left(1-\frac{C_{p}}{C_{f}}\right)\times 100\%$$

where *R* is the salt rejection of membranes, %; *C_f_* is the salt concentration of the feed solution, ppm; and *C_p_* is the salt concentration of the permeate solution, ppm.

## 3. Results and Discussion

### 3.1. Surface Properties of Membranes

**Figure 2:**
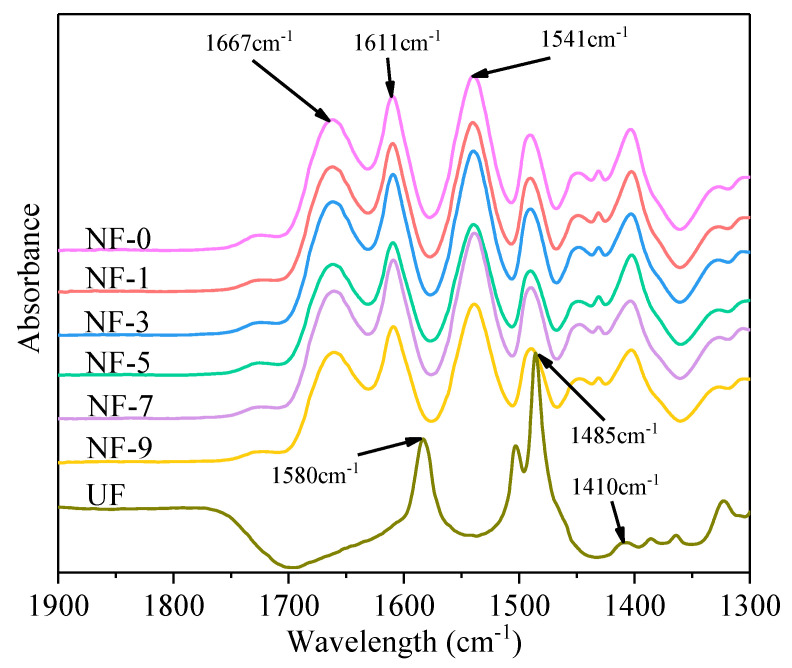
Infrared spectrum of membrane with different DWCNT dosages.

The chemical groups of the NF membranes’ surface were characterized by ATR-FTIR. The PES substrate has three characteristic spectra at 1410 cm^−1^, 1485 cm^−1^ and 1580 cm^−1^ (Figure 2) due to aromatic ring (benzene) vibration [28,57]. However, for the composite NF membranes, three new peaks at 1541 cm^−1^, 1611 cm^−1^ and 1667 cm^−1^ related to C=O stretching of the amide I bond, aromatic amide ring breathing and N–H bending of amide II in the –CO–NH– group [58,59], respectively, were identified in Figure 2. The three characteristic peaks indicated that the polyamide functional layer had been successfully prepared on the surface of the PES substrate by the interfacial polymerization method. In addition, Figure 2 shows that the addition of DWCNT has little effect on the infrared peak of NF membranes, which indicated that new functional groups were not generated on the surface of NF membranes after the addition of DWCNT.

An XPS analysis was carried out to characterize the elemental composition of the polyamide layer (5~10 nm in the surface layer) [60]. The XPS analysis confirmed that there was no characteristic element S on the surface of the NF membrane, indicating that the PES substrate surface was completely covered by a polyamide layer with a thickness of more than 10 nm. The crosslinking degree can be calculated in terms of the atomic concentration of the membrane surface. The calculation formula is as follows [60]:

(4) $$\mathrm{Crosslinking}\;\mathrm{Degree}\left(\%\right)=\frac{X}{X+Y}\times 100\%$$

where *X* and *Y* represent cross-linked and linear parts, respectively (Figure 1). The crosslinking degree can be calculated according to the atomic ratio of N and O [61]:

(5) $$\frac{O}{N}=\frac{3X+4Y}{3X+2Y}$$

(6) $$\mathrm{Crosslinking}\;\mathrm{Degree}\left(\%\right)=\frac{4N-2O}{N+O}\times 100\%$$

**Figure 3:**
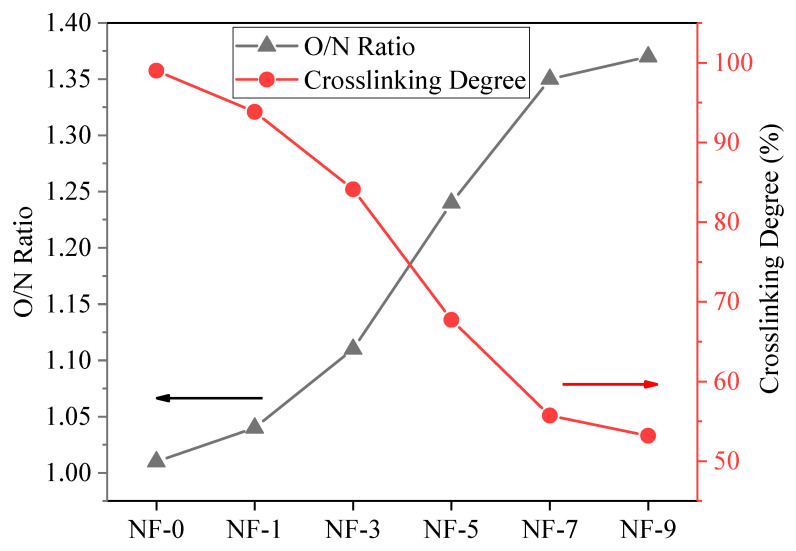
O/N ratio and crosslinking degree of NF membranes with different DWCNT dosages.

In theory, the range of the crosslinking degree is 0~100%. When the crosslinking degree is 100%, the polyamide completely forms a cross-linked structure, and thus *O*/*N* is equal to 1; when the crosslinking degree is 0, the whole structure is linear, and the *O*/*N* is equal to 2. The atomic concentration, *O*/*N* ratio and crosslinking degree of the polyamide layer are shown in Figure 3. It can be seen that with the increase of the DWCNT dosage, the *O*/*N* ratio increased gradually, which indicated that the linear structure increased and that the crosslinking degree of the polyamide layer decreased. Figure 3 illustrates that the addition of DWCNT led to the reduction of amide groups and the increase of carboxyl groups [62]. This phenomenon can be explained by the diffusion and migration of MPD in the reaction region. When the substrate was immersed in the aqueous solution, the MPD monomer entered the substrate pores and adhered to the surface of the substrate. Additionally, when the substrate was immersed in TMC solution, the MPD diffused to the reaction zone and reacted with TMC, after which polyamide was formed in the substrate pores and on the surface of the substrate [63]. At the same time, some water molecules adhered to the substrate surface with the addition of DWCNT because of its strong hydrophilicity. During the interfacial polymerization reaction, water molecules diffused to the reaction zone, leading to the hydrolysis reaction of TMC and formation of more carboxyl groups, and the crosslinking degree of NF membranes decreased.

**Figure 4:**
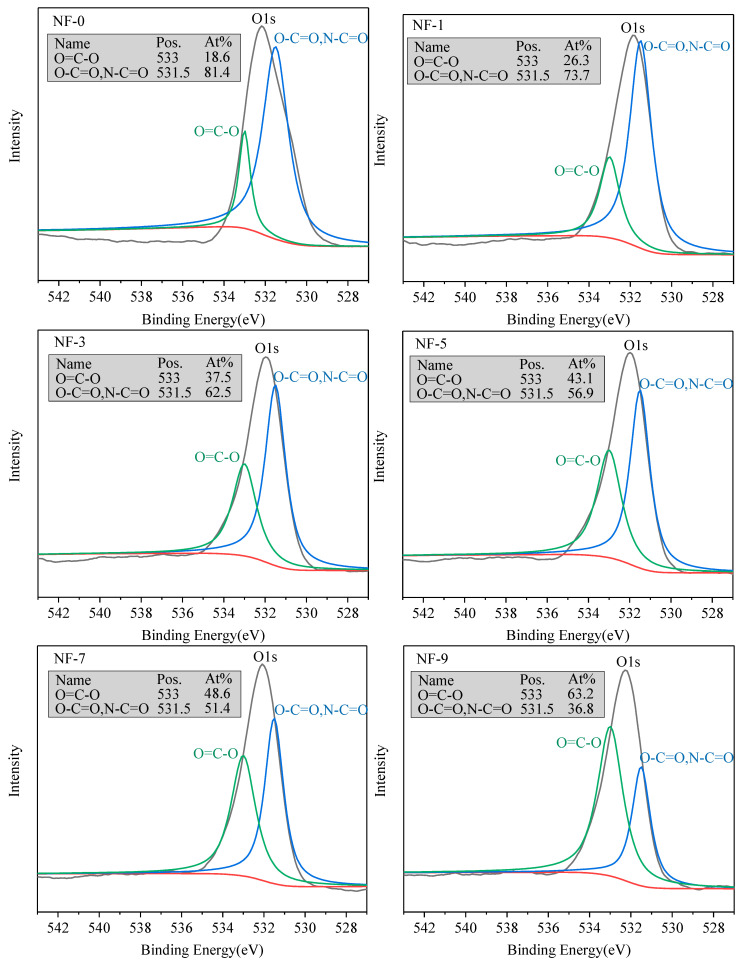
Oxygen 1s XPS spectra of NF membranes with different DWCNT dosages.

The XPS can also test the ratio of oxygen in different functional groups. The O1s has two peaks at binding energies of 531.5 eV and 533 eV, representing carbonyl oxygen bonding (O=C-O/N-C=O) and carboxyl oxygen bonding (O=C-O) [64], respectively. As seen in Figure 4, the ratio of carboxyl oxygen (At%) rose with the DWCNT dosage increase, which indicated that the crosslinking degree decreased and that the number of carboxyl groups on the NF membrane surface increased.

**Figure 5:**
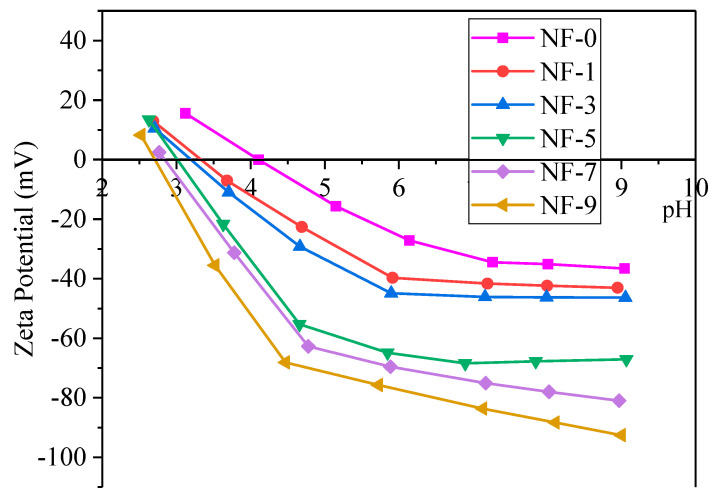
Zeta potential of NF membranes with different DWCNT dosages.

Zeta potential is used to characterize the electric charge on the membrane surface [21]. The variation trend of membranes’ zeta potential for different DWCNT dosages is shown in Figure 5. The isoelectric point (IEP) decreased with the increase of the DWCNT dosage, from 4.2 without DWCNT modification to 2.7 for NF membranes modified with 9 ml DWCNT dispersion, which indicated that the addition of DWCNT increased the negative electric charge on the membrane surface [49]. In this experiment, it showed that the number of carboxyl groups on the membrane surface increased, which was consistent with the XPS test results.

**Figure 6:**
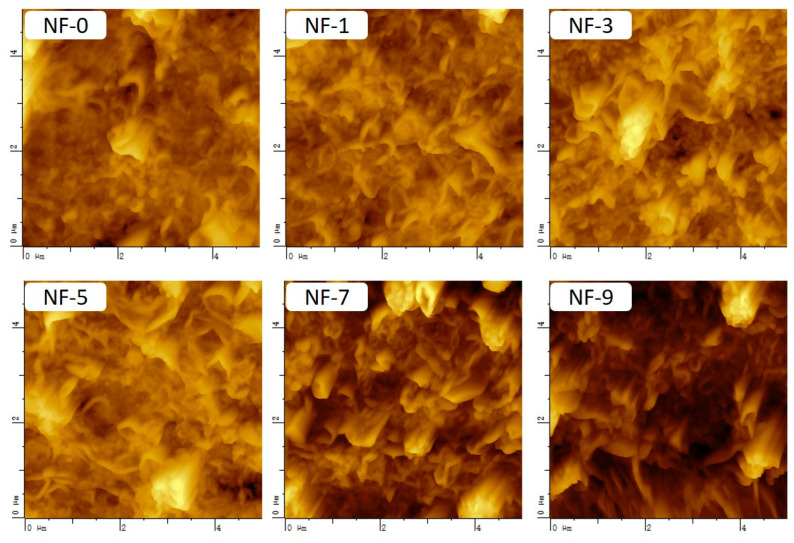
AFM of NF membranes prepared with different DWCNT dosages.

The surface morphology of DWCNT-modified membranes characterized by AFM is shown in Figure 6 exhibiting a lotus-leaf-like structure. Additionally, with the increase of the DWCNT dosage, peak-valley structures appeared more obviously, and the mean roughness (Ra) and root mean square (Rms) of the polyamide layer gradually increased (Table 1). Because most DWCNTs cannot enter into the pore channels but adhere to the substrate surface, which enlarges the height difference of the substrate surface, and because the roughness of membranes is positively correlated with the height difference of the substrate surface, the roughness of NF membranes increased with the addition of DWCNT. Meanwhile, the specific surface area of NF membranes gradually increased as well, which was beneficial to improving the water flux of NF membranes.

**Figure 7:**
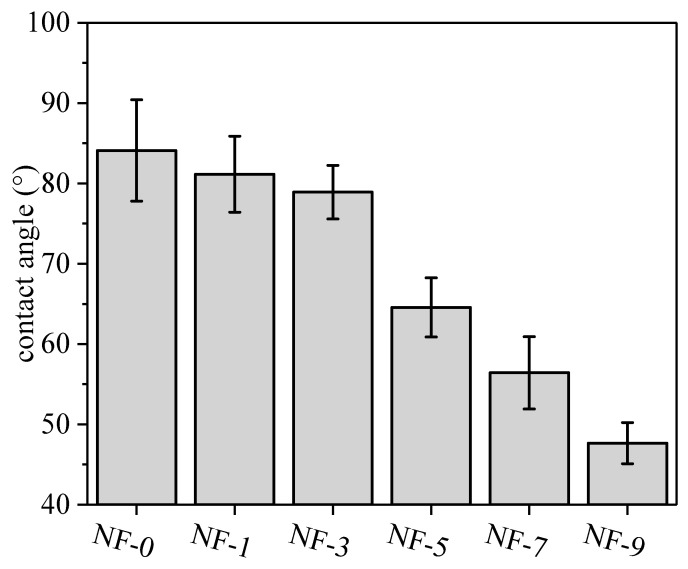
Contact angle of NF membranes with different DWCNT dosages.

The water contact angle is an important parameter to evaluate the wettability. In general, the increase of roughness can decrease the contact angle of hydrophilic materials but increase the contact angle of hydrophobic materials [4,65]. As shown in Figure 7, the contact angle of NF membranes decreased significantly with the increase of the DWCNT dosage, which was related to the changes of functional groups and the roughness of the membrane surface. According to the analysis of the XPS and zeta potential results, the addition of DWCNT could increase the number of carboxyl groups on the membrane surface, which improved the hydrophilicity of the membrane. Moreover, the roughness also increased with the addition of DWCNT. Under the influence of functional groups and the surface morphology, the contact angle of membranes decreased with the addition of DWCNT.

### 3.2. Membrane Performance Tests

**Figure 8:**
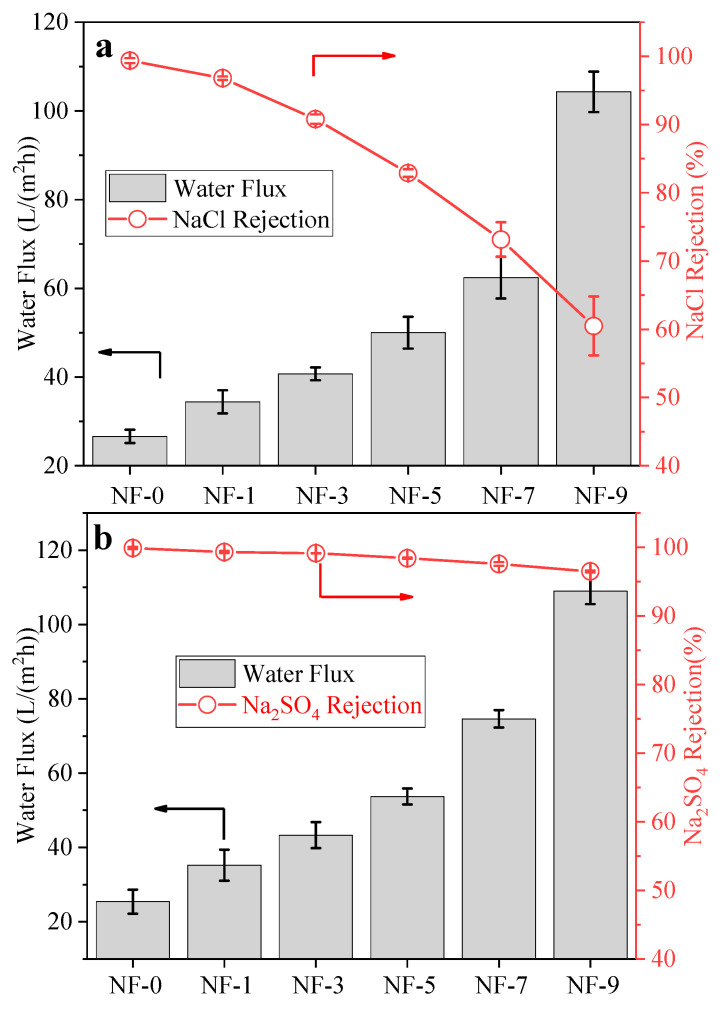
Water flux and rejection of (**a**) NaCl/(**b**) Na_2_SO_4_ with different DWCNT dosages.

The water permeability and salt rejection of NF membranes were evaluated and are shown in Figure 8. Generally, the growing DWCNT dosage increased the water flux of membranes but decreased the salt rejection. For NaCl and Na_2_SO_4_ solutions, the water flux increased from 26.6 L/(m^2^·h) and 25.4 L/(m^2^·h) to 104.3 L/(m^2^·h) and 109 L/(m^2^·h), while the salt rejection decreased from 99.38% and 99.88% to 60.47% and 96.48%, respectively. The phenomenon of water flux increasing with the DWCNT dosage could be attributed to the following three factors. Firstly, according to the results of the XPS, zeta potential and contact angle, the addition of DWCNT increased the quantity of carboxyl groups of the NF membranes, which improved the hydrophilicity of the membrane surface [4], and then in turn reduced the resistance of water molecules passing through the boundary layer to the membrane surface, thus decreasing the overall resistance of NF membranes. Secondly, the addition of DWCNT increased the specific surface area, which provided more penetration sites for water molecules passing through the polyamide layer and improved the transmission efficiency [66,67]. Thirdly, the water molecules with a diameter of approximately 0.4 nm can, at 25 °C [68], pass through the inner channels of DWCNT. Like the “water channel” between the polyamide layer and the substrate, internal channels of DWCNT facilitated the water molecules entering into substrate pores, which reduced the penetration resistance of water molecules. At the same time, the channels formed between the polyamide and DWCNT also provided another path for water molecules.

There are two reasons for the difference in the rejection rate of NaCl and Na_2_SO_4_. With the increase of the DWCNT dosage, the quantity of carboxyl groups on the membrane surface had a sharp increase. Due to the Donnan exclusion effect, the repulsive forces between divalent sulfate ions were much larger than those of chloride ions [49]. Moreover, the increase of the DWCNT dosage gradually decreased the crosslinking degree of the polyamide layer [51], and the hydrated ionic radius of sulfate ions was greater than that of chloride ions. Therefore, under the joint influence of size sieving and the Donnan exclusion effect [4,69,70], sulfate ions were more difficult to pass through the membranes. Hence, the rejection rate of Na_2_SO_4_ was much higher than that of NaCl.

## 4. Conclusions

In this study, NF membranes were prepared by the interfacial polymerization method on a PES substrate modified with DWCNT. By adjusting the dosage of DWCNT, NF membranes with different crosslinking degrees and roughness values were prepared. Additionally, due to the changes of the membrane surface properties and the internal structure of the polyamide layer, the water flux increased by about three times. For 2000 ppm NaCl and Na_2_SO_4_ solutions, the water flux increased from 26.6 L/(m^2^·h) and 25.4 L/(m^2^·h) to 104.3 L/(m^2^·h) and 109 L/(m^2^·h), respectively, while the salt rejection decreased from 99.38% and 99.88% to 60.47% and 96.48%. The modified membranes exhibit a high rejection rate of Na_2_SO_4_ due to size sieving and the Donnan exclusion effect. These findings propose an avenue to fabricate high-flux NF membranes.

## Figures and Tables

**Table 1:** Surface parameters of NF membranes with different DWCNT dosages.

	Specific Surface Area (S/P)	Roughness	
		Ra/nm	Rms/nm
NF-0	1.68	76.3	97.2
NF-1	1.74	79.6	101.1
NF-3	1.82	85.9	109.9
NF-5	1.95	94.1	120.1
NF-7	2.06	103.5	132.6
NF-9	2.11	105.4	135.1

## Data Availability

Not applicable.
